# Chondrogenic differentiation potential of osteoarthritic chondrocytes and their possible use in matrix-associated autologous chondrocyte transplantation

**DOI:** 10.1186/ar2800

**Published:** 2009-09-02

**Authors:** Tilo Dehne, Camilla Karlsson, Jochen Ringe, Michael Sittinger, Anders Lindahl

**Affiliations:** 1 Tissue Engineering Laboratory and Berlin-Brandenburg Center for Regenerative Therapies, Department of Rheumatology and Clinical Immunology, Charité-Universitätsmedizin Berlin, Tucholskystraße 2, Berlin, 10117, Germany; 2 Institute of Laboratory Medicine, Department of Clinical Chemistry and Transfusion Medicine, Sahlgrenska University Hospital, Bruna Stråket 16, Gothenburg, SE 413-45, Sweden

## Abstract

**Introduction:**

Autologous chondrocyte transplantation (ACT) is a routine technique to regenerate focal cartilage lesions. However, patients with osteoarthritis (OA) are lacking an appropriate long-lasting treatment alternative, partly since it is not known if chondrocytes from OA patients have the same chondrogenic differentiation potential as chondrocytes from donors not affected by OA.

**Methods:**

Articular chondrocytes from patients with OA undergoing total knee replacement (Mankin Score > 3, Ahlbäck Score > 2) and from patients undergoing ACT, here referred to as normal donors (ND), were isolated applying protocols used for ACT. Their chondrogenic differentiation potential was evaluated both in high-density pellet and scaffold (Hyaff-11) cultures by histological proteoglycan assessment (Bern Score) and immunohistochemistry for collagen types I and II. Chondrocytes cultured in monolayer and scaffolds were subjected to gene expression profiling using genome-wide oligonucleotide microarrays. Expression data were verified by using real-time PCR.

**Results:**

Chondrocytes from ND and OA donors demonstrated accumulation of comparable amounts of cartilage matrix components, including sulphated proteoglycans and collagen types I and II. The mRNA expression of cartilage markers (*ACAN, COL2A1, COMP, CRTL1, SOX9*) and genes involved in matrix synthesis (*BGN*, *CILP2, COL9A2, COL11A1, TIMP4*) was highly induced in 3D cultures of chondrocytes from both donor groups. Genes associated with hypertrophic or OA cartilage (*ALPL, COL1A1, COL3A1, COL10A1, MMP13, POSTN, PTH1R, RUNX2*) were not significantly regulated between the two groups of donors. The expression of 661 genes, including *COMP*, *FN1*, and *SOX9*, was differentially regulated between OA and ND chondrocytes cultured in monolayer. During scaffold culture, the differences diminished between the OA and ND chondrocytes, and only 184 genes were differentially regulated.

**Conclusions:**

Only few genes were differentially expressed between OA and ND chondrocytes in Hyaff-11 culture. The risk of differentiation into hypertrophic cartilage does not seem to be increased for OA chondrocytes. Our findings suggest that the chondrogenic capacity is not significantly affected by OA, and OA chondrocytes fulfill the requirements for matrix-associated ACT.

## Introduction

The regenerative capacity of articular cartilage is very limited and injuries that do not penetrate the subchondral bone do not self-repair in adults. This low potential for regeneration has resulted in the development of a number of techniques intended to restore hyaline cartilage defects [1]. One treatment option is autologous chondrocyte transplantation (ACT) developed by Brittberg and colleagues in the early 1990s [2]. This technique is based on the isolation of chondrocytes from a minor load-bearing area of the knee, cell expansion and re-transplantation as cell suspensions. This first generation of cell-based treatment has been followed by a second generation, consisting of culture-expanded chondrocytes seeded into a biodegradable scaffold before implantation [3-5].

Today, esterified hyaluronic acid-based scaffolds, collagen membranes and gels, and fibrin-polymer scaffolds are used as delivery vehicles for second generation ACT. These scaffolds are resorbed *in vivo *allowing complete replacement of the implant with newly formed tissue and also support re-differentiation of the chondrocytes [3,5-7]. Advantages of this second-generation technique include a more uniform distribution of the cells and prevention of cells escaping into the articular cavity. Another advantage is the potential for treating larger defects [8]. This is of special importance for patients with osteoarthritis (OA), who today are lacking an appropriate long-lasting treatment alternative [9].

Several articles have demonstrated phenotypical alterations in OA chondrocytes *in vivo *compared with normal chondrocytes. The expression of genes belonging to hypertrophic cartilage (*collagen type X*) and more primitive cartilage (*collagen type I *and *collagen type III*) was increased, while the expression of genes characteristic for a mature articular cartilage phenotype was significantly decreased (*aggrecan*,*cartilage link protein 1*,*SRY (sex determining region Y)-box 9*) in comparison with normal cartilage [10,11]. Some articles reported that these OA-related alterations influence bioactivity and matrix gene expression negatively when cultured *in vitro *[12,13]. Others demonstrated that OA chondrocytes display a good proliferation potential and were able to re-differentiate resulting in a matrix rich in proteoglycans and collagen type II [14,15]. Such conflicting data encouraged us to investigate more thoroughly the chondrogenic potential of OA chondrocytes for possible use in second-generation ACT.

In this study, the chondrogenic capacity of expanded chondrocytes from normal and OA donors was examined comparatively to investigate whether OA chondrocytes are suited for cartilage tissue engineering approaches in OA. Therefore, protocols as used for ACT were applied for chondrocyte preparation and expansion. The differentiation potential was histologically analyzed after 14 days in high-density pellet and hyaluronan-based scaffold cultures. Aiming on a comprehensive molecular analysis of the differentiation process of OA chondrocytes, expanded chondrocytes and chondrocytes in scaffold cultures were subjected to gene expression profiling using genome-wide Affymetrix oligonucleotide microarrays.

## Materials and methods

### Biopsy collection and Mankin scoring

**Figure 1:**
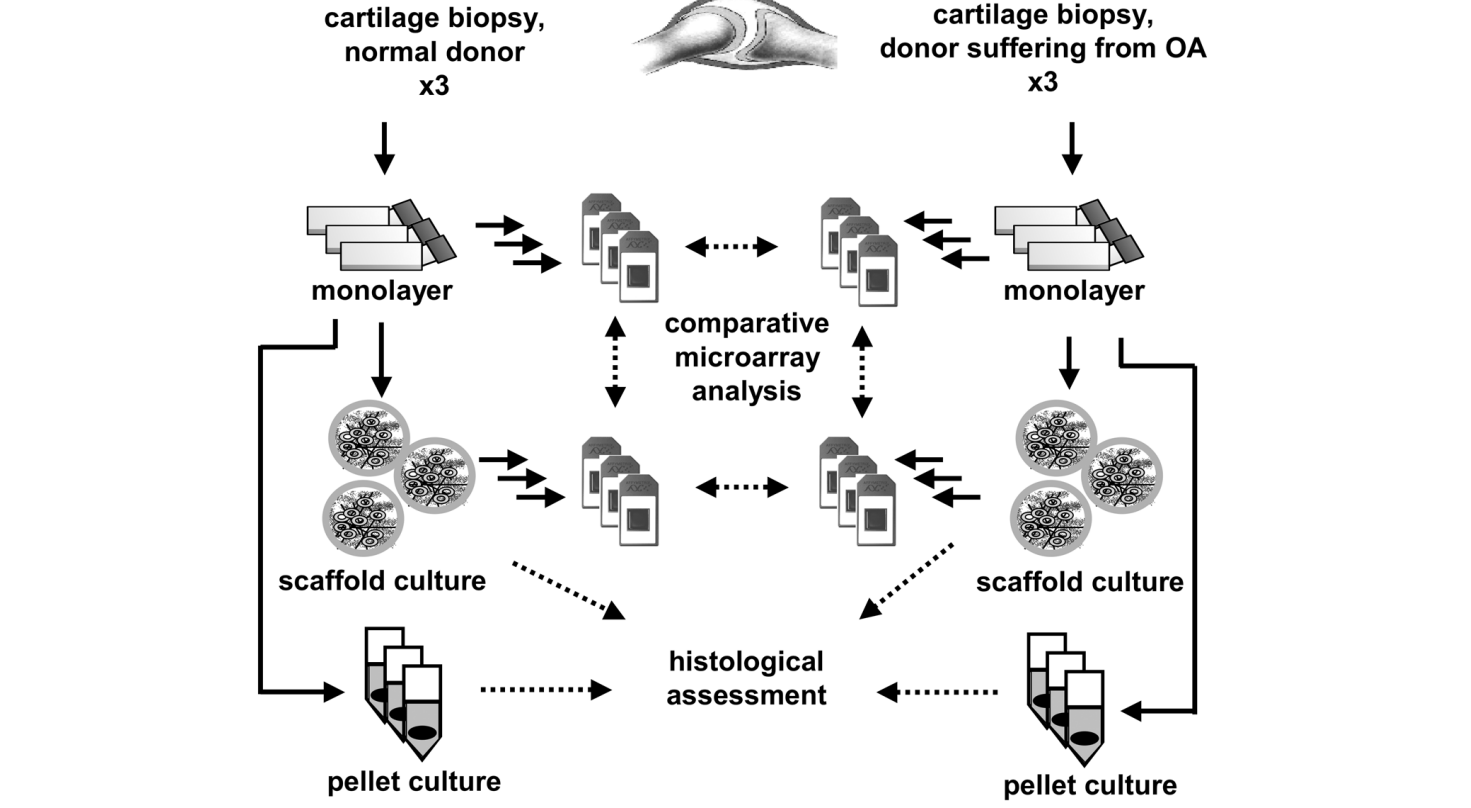
Schematic illustration of experimental setup.  Articular chondrocytes from three patients with osteoarthritis and from three patients undergoing autologous chondrocyte transplantation (ACT) were isolated applying protocols used for ACT. After expansion in monolayer the chondrogenic differentiation potential was evaluated in high-density pellet and scaffold (Hyaff-11) cultures by histological assessment (Bern Score, immunohistochemistry for collagen types I and II). Chondrocytes cultured in monolayer and scaffolds were subjected to comparative gene expression analysis (genome-wide oligonucleotide microarrays, real-time PCR).

Patients with OA were selected for the study if they fulfilled five criteria: symptoms of severe OA, undergoing total knee replacement, radiological evidence of OA, OA grade 2 to 3 according to Ahlbäck score, and exhibiting a Mankin score above 3. Articular cartilage from three donors (one female and two males) was collected based on these criteria. The donors age ranged from 60 to 64 years (average 62 years) with a Mankin score of 3 to 7. Control patients were selected for inclusion in the study if they had no pre-existing history of OA symptoms, macroscopically healthy cartilage, and were undergoing ACT treatment (these donors are referred to as normal donors (ND)). ND articular cartilage biopsies were obtained from three donors (age range 46 to 52 years, average age 50 years, one female and two males). The biopsies were transported to the cell culture laboratory in sterile saline solution (0.9% sodium chloride; Fresenius Kabi, Uppsala, Sweden) supplemented with gentamicin sulphate (50 mg/l; Gibco, Paisley, Renfrewshire, UK) and amphotericin B (250 μg/ml; Gibco, Paisley, Renfrewshire, UK). One part of each OA cartilage biopsy was processed for histology, stained with Safranin-O and Alcian Blue van Gieson, blinded and scored in accordance with a modified (biopsies without subchondral bone) Mankin scale, with a maximum score of 13. All six donors were used to carry out the following investigations (Figure 1). The donation of cartilage was approved by the ethical committee at the Medical Faculty at Gothenburg University (ethical permission number S 040-01). Informed consent had been obtained from cartilage donors.

### Cell culture and chondrogenic differentiation

Primary chondrocytes were isolated from the surrounding matrix as described previously [2]. The isolated cells were seeded at 10^4^cells/cm^2 ^in culture flasks (cell passage 0; Costar; Corning Incorporated, Corning, NY, USA) in expansion medium consisting of DMEM/Ham's F12 (Gibco, Paisley, Renfrewshire, UK) supplemented with L-ascorbic acid (0.025 mg/ml; Apotekets production unit, Umeå, Sweden), gentamicin sulphate (50 mg/l; Gibco, Paisley, Renfrewshire, UK), amphotericin B (250 μg/ml; Gibco, Paisley, Renfrewshire, UK) and L-glutamine (2 mM; Gibco, Paisley, Renfrewshire, UK) and 10% human serum.

In order to induce chondrogenesis, cells in passage 2 were cultured in either high-density pellet cultures or hyaluronan-based biodegradable polymer scaffolds (Hyaff-11) developed for tissue- engineering applications, as described previously [15]. For pellet mass cultures, 2 × 10^5 ^cells in passage 2 were placed into a conical polypropylene tube with 0.5 ml of defined medium, consisting of DMEM high glucose (PAA Laboratories, Linz, Austria) supplemented with 5.0 μg/ml linoleic acid (Sigma-Aldrich, Stockholm, Sweden), insulin-transferrin-selenium-G (ITS-G; Gibco, Paisley, Renfrewshire, UK), 1.0 mg/ml human serum albumin (Equitech-Bio, Kerrville, TX, USA), 10 ng/ml transforming growth factor beta 1 (TGF-β1; R&D Systems, Abingdon, UK), 10^-7 ^M dexamethasone (Sigma-Aldrich, Stockholm, Sweden), 14 μg/ml L-ascorbic acid (Apotekets, Umeå, Sweden) and 1% penicillin-streptomycin (PEST, PAA Laboratories, Linz, Austria). The cells were centrifuged at 500 g for five minutes and maintained in 37°C in 7% carbon dioxide/93% air with medium changes twice a week. For scaffold culture, 2 × 10^6 ^cells/cm^2 ^were seeded in Hyaff-11 scaffolds, 4 cm^2 ^in size (Fidia Advanced Biopolymers, Abano Terme, Italy), pre-coated with human serum.

After 14 days of chondrogenic differentiation, the specimens were fixed in Histofix™ (Histolab products AB, Gothenburg, Sweden), dehydrated with ethanol, and embedded in paraffin. Five-micrometer sections were cut and placed onto silane-coated glass slides (Superfrost Plus, Menzel-Gläser, Germany). The sections were deparaffinized and stained with Alcian Blue van Gieson and Safranin-O, and were then observed with a light microscope (Nikon, Tokyo, Japan). Chondrogenesis was further analyzed using the Bern Score as described previously [16]. Briefly, this scoring system assesses the uniformity and intensity of matrix staining, cell density/extent of matrix produced, and cellular morphologies, which is graded according to the Bern Score scale. The results for the single observations of each assessed ND and OA sample were averaged and used for statistical analysis. Differentiation was also studied by immunohistochemical localization of collagen types I and II as described below.

### Immunohistochemistry

The expression of collagen types I and II was studied in both pellet and scaffold cultures. Sections of the pellets were deparaffinized, dehydrated, digested with 8000 U/ml hyaluronidase (Sigma-Aldrich, Stockholm, Sweden) in PBS for one hour at 37°C and blocked with 3% BSA (Sigma-Aldrich, Stockholm, Sweden). Then, sections were labeled with primary monoclonal antibodies raised against collagen types I and II (anti-collagen type I and II (ICN Biomedicals, Aurora, OH, USA)) diluted 1:150. Subsequently, primary antibodies were visualized using a horseradish peroxidase-conjugated secondary antibody (goat-anti-mouse) (Jackson Laboratory, Maine, ME, USA), diluted 1:150. All incubations were performed at room temperature in a humidified chamber for one hour. Horseradish peroxidase, and therefore also the secondary antibodies, were visualized using the TSA-Direct Cy3 kit (Perkin Elmer, Boston, MA, USA) according to the manufacturer's instructions. Nuclei were stained with 4',6-Diamidino-2-phenylindol (Sigma-Aldrich, Stockholm, Sweden) and the slides were mounted in antifading medium. The sections were then analyzed using a fluorescence microscope (Nikon, Tokyo, Japan) and digital pictures were taken with the ACT-1 software (Nikon, Tokyo, Japan). Positive controls were sections from goat hyaline cartilage obtained from the knee and negative controls were sections incubated with only secondary antibody.

### RNA isolation

Total RNA from chondrocytes cultured in monolayer (ML; passage 2) was isolated applying protocols for animal tissues of the RNeasy Mini Kit (Qiagen, Hilden, Germany). For scaffold cultures, an 8 mm punch was prepared, snap-frozen in liquid nitrogen, and stored at -80°C until further use. Frozen samples were transferred to 1 ml TriReagent (Sigma-Aldrich, Stockholm, Sweden) and mechanically homogenized. Subsequently, 133 μl 1-Bromo-3-chloro-propane (Sigma-Aldrich, Stockholm, Sweden) was admixed followed by centrifugation for 45 minutes at 13,000 g. The aqueous phase was collected and nucleic acids were precipitated by addition of an equal volume of ice-cold isopropanol. After 30 minutes incubation the precipitated nucleic acids were pelleted and resolved in 350 μl RLT buffer (Qiagen, Hilden, Germany). Further purification was performed according to protocols for animal tissues of the RNeasy Mini Kit (Qiagen, Hilden, Germany).

### Microarray analysis

RNA from ML and scaffold cultures was subjected to gene expression analysis using oligonucleotide microarray HG-U133plus2.0 (Affymetrix, Santa Clara, CA, USA) according to the manufacturer's recommendations. Briefly, 2 μg of total RNA were used to synthesize biotin-labeled cRNA. Ten microgram samples of fragmented cRNA were hybridized to GeneChips for 16 hours at 45°C. Washing, staining and scanning of the microarrays were performed using the Affymetrix GeneChip equipment (Santa Clara, CA, USA). Raw expression data were normalized and subsequently analyzed with the GeneChip Operating Software 1.4 (GCOS, Affymetrix, Santa Clara, CA, USA). For comparative analysis the workflow implemented in the SiPaGene database was applied [17]. In detail, samples of each scaffold culture (three-dimensional (3D)) were compared with ML cultures as baseline, for OA and ND separately. Furthermore, OA ML and 3D cultures were compared with corresponding ND cultures as baseline (for schematic illustrations of comparative analysis see Figure 1). Genes were regarded as differentially regulated when fulfilling specific change call criteria. The limit was set to at least eight (of nine possible) significant change calls. Functional classification was conducted with annotations from the Gene Ontology Annotation Database [18]. Expression differences were given as fold changes (FC). The significance level was determined applying the Welch's t-test on log2-transformed signal values. Hierarchical cluster analysis was performed with log2-transformed signals normalized by genes and Pearson correlation as distance measure using Genesis 1.7.2 software (Graz University of Technology, Institute for Genomics and Bioinformatics, Graz, Austria) [19]. Microarray data have been deposited in the National Center for Biotechnology Information Gene Expression Omnibus and are accessible through Gene Expression Omnibus series accession number [GSE16464].

### Real-time PCR

Equal amounts of the remaining RNA not used for microarray analysis were reverse transcribed with the iScript cDNA synthesis kit (BioRad, München, Germany). cDNA was amplified using SYBR green PCR reagents (Applied Biosystems, Darmstadt, Germany) and the iCycler (BioRad, München, Germany). The expression of *glyceraldehyde-3-phosphate dehydrogenase *(*GAPDH*) was used to normalize samples by adjusting the sample cDNA concentration. Marker gene expression (Table 1) is given as a percentage related to *GAPDH *expression [20].

**Table 1:** Primer oligonucleotide sequences used for real-time PCR

Gene	Forward primer 5'-3'	Reverse primer 5'-3'	Accession number
COL1A1	CGATGGCTGCACGAGTCACAC	CAGGTTGGGATGGAGGGAGTTTAC	[GenBank:NM_000088]
COL10A1	GAACTCCCAGCACGCAGAATCC	GTGTTGGGTAGTGGGCCTTTTATG	[GenBank:NM_000493]
COL2A1	CCGGGCAGAGGGCAATAGCAGGTT	CATTGATGGGGAGGCGTGAG	[GenBank:NM_001844]
COMP	GGGTGGCCGCCTGGGGGTCTT	CTTGCCGCACGCTGATGGGTCTC	[GenBank:NM_000095]
CRTL1	GCGTCCGCTACCCCATCTCTA	GCGCTCTAAGGGCACATTCAGTT	[GenBank:NM_001884]
GAPDH	GGCGATGCTGGCGCTGAGTAC	TGGTTCACACCCATGACGA	[GenBank:NM_000095]
MMP1	TACATGCGCACAAATCCCTTCTACC	GAAAAACCGGACTTCATCTCTGTCG	[GenBank:NM_002421]
MMP13	CAAAAACGCCAGACAAATGTGACC	GATGCAGGCGCCAGAAGAATCT	[GenBank:NM_002427]
SOX9	CTGAGTCATTTGCAGTGTTTTCT	CATGCTTGCATTGTTTTTGTGT	[GenBank:NM_000346]
TIMP4	TTTCTTCTGGCTTAGTCTGTTTTCT	ATTCGCCATTTCTCCCCTACCA	[GenBank:NM_003256]

## Results

### Histology and immunohistochemistry

**Figure 2:**
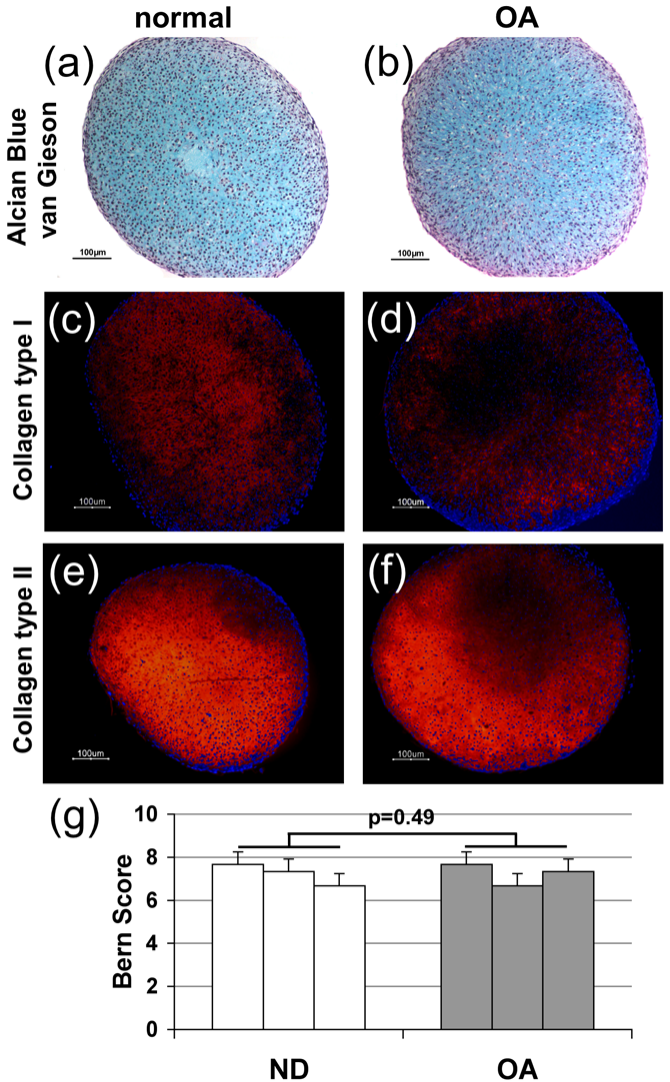
Histology of normal donor and osteoarthritic chondrocyte pellet cultures.  Chondrogenic differentiation of chondrocytes obtained from **(a, c, e) **normal donors (ND) and **(b, d, f) **osteoarthritic (OA) articular cartilage using the high-density pellet culture system. **(a, b) **Alcian Blue van Gieson staining and immunohistochemical localization of **(c, d) **collagen type I and **(e, f) **type II. **(g) **Bern Score evaluating the differentiation grade of the cells. Three cultures per donor group.

**Figure 3:**
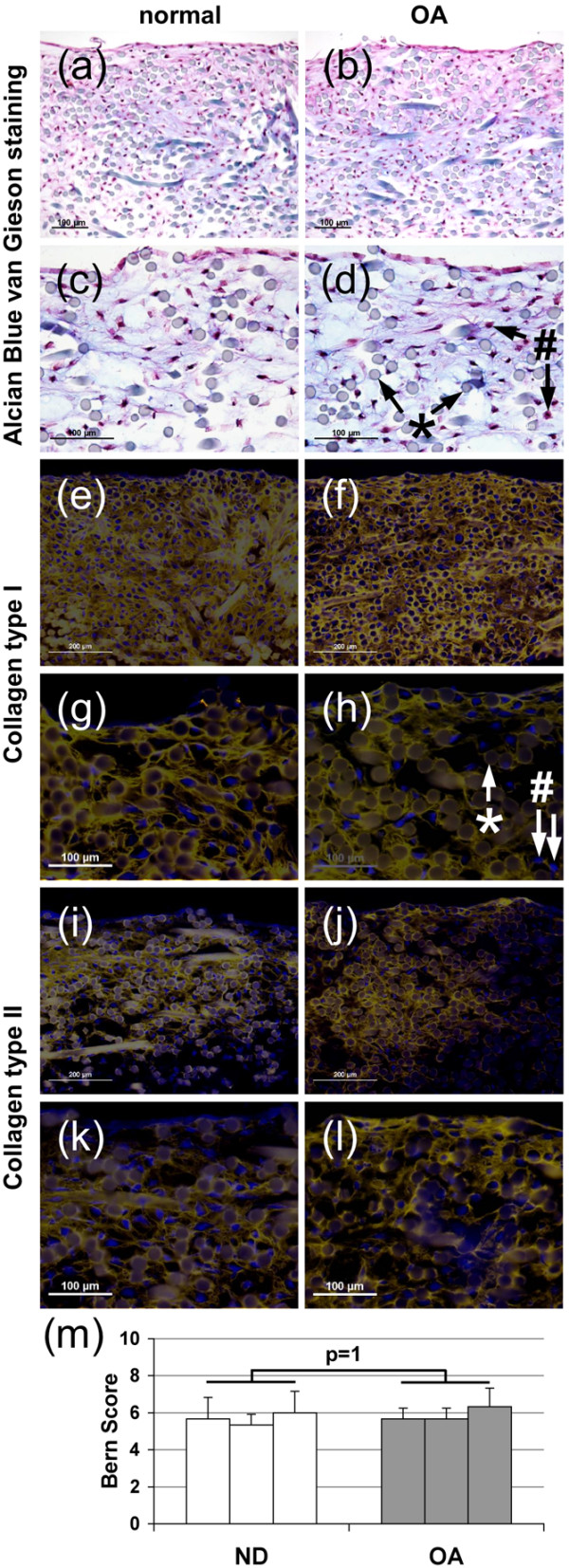
Histology of osteoarthritic and normal chondrocyte scaffold culture.  Chondrogenic differentiation of chondrocytes obtained from **(a, c, e, g, i, k) **normal and **(b, d, f, h, j, l) **osteoarthritic (OA) articular cartilage cultured in Hyaff-11 scaffolds. **(a to d) **Alcian Blue van Gieson staining, immunohistochemical localization of collagen **(e to h) **type I and **(I to l) **type II, with **(g, h, k, l) **higher magnification, and **(m) **Bern Score, * scaffold fibre, # cell nuclei. Three cultures per donor group.

After 14 days of differentiation, intense Alcian Blue van Gieson staining was detected in pellets from both ND (Figure 2a) and OA (Figure 2b) chondrocytes, demonstrating accumulation of sulphated proteoglycans. A matrix containing collagen types I (Figures 2c, d) and II (Figures 2e, f) was detected in these pellets, but no differences were detected between ND (Figures 2c, e) and OA (Figures 2d, f) chondrocytes. Additionally, applying the Bern Score system for histological assessment of the pellets demonstrated that there were no significant differences in the cartilage quality between OA and ND chondrocytes (Figure 2g). A less differentiated phenotype was detected in the scaffold-cultured cells, but accumulation of sulphated proteoglycans was still detected using Alcian Blue van Gieson in ND (Figures 3a, c) and OA (Figures 3b, d) cultures. No significant differences in accumulation of a cartilaginous matrix could be detected between OA and ND chondrocytes cultured in scaffolds applying the Bern Score (Figure 3m). Accumulation of both collagen types I (Figures 3e to 3h) and II (Figures 3i to 3l) was detected in Hyaff-11 scaffolds seeded with either healthy (Figures 3e, g, i, k) or OA (Figures 3f, h, j, l) chondrocytes, no significant differences were detected between the two cell sources. In accordance with the Alcian Blue van Gieson staining, less accumulation of collagen type II was detected in the Hyaff-11 scaffolds compared with the high-density pellet cultures.

### Comparative gene expression analysis

Comparative microarray analysis identified a total number of 1336 genes that were differentially regulated comparing ND chondrocytes cultured in monolayer and scaffold culture, while 2534 genes were regulated making the same comparison for OA chondrocytes (Table 2) [see Additional data file 1]. Fewer genes were regulated comparing OA and ND chondrocytes cultured in ML (661 genes regulated) and scaffold culture (184 genes regulated). Further examination was performed on the basis of genes associated with differentiation processes, which were identified with annotations obtained from the Gene Ontology Database (terms 'skeletal development' and 'extracellular matrix (ECM) formation) [see Additional data file 2]. This resulted in a selection of genes coding for collagens, proteoglycans, matrix-modifying enzymes, cell attachment components, growth factors, surface receptors, and transcription factor. Initially, the expression profiles of ND chondrocytes during ML culture (baseline) and Hyaff-11 culture were generated and compared. Secondly, significantly regulated genes obtained in the initial analysis were used as reference to study OA chondrocytes cultured in ML and scaffolds.

**Table 2:** Overview of number of genes differentially expressed in chondrocyte monolayer and scaffold culture

	3D vs ML		OA vs ND	
Significance level	ND	OA	ML	3D
GCOS	107 (1336)	152 (2534)	32 (661)	17 (184)
+ *P *< 0.05	60 (724)	110 (1723)	7 (331)	1 (12)
+ *P *< 0.01	24 (217)	43 (613)	0 (78)	0 (1)
+ *P *< 0.001	3 (27)	8 (92)	0 (10)	0 (0)

Comparisons between scaffold (3D) and monolayer (ML) cultures were performed for chondrocytes obtained from osteoarthritic (OA) and normal donors (ND) (see Figure 1 for experimental setup). Genes were functionally filtered by annotations of the Gene Ontology Database according to their association with skeletal development and extracellular matrix formation [see Additional data file 2 for full list]. Genes were regarded as differentially expressed when fulfilling specific change call criteria provided by GeneChip Operating Software (GCOS, Affymetrix). The limit was set to at least eight (of nine possible) significant change calls. Further significance levels were determined applying the Welch's t-test of the SiPaGene database [17]. Numbers in brackets represent the total number of genes regulated without functional filtering [see Additional data file 1 for full list].

### Gene expression profiling during normal donor differentiation

One hundred and seven genes were found differentially expressed comparing ND scaffold cultures with ND chondrocytes cultured in ML (baseline) [see Additional data file 2]. Scaffold culture resulted in a significantly increased expression of cartilage markers such as *collagen type IIα1 (COL2A1) *and *cartilage oligomeric matrix protein (COMP)*, about 80-fold and 120-fold, respectively (Table 3). Expression of the proteoglycans *aggrecan (ACAN) *and *cartilage link protein 1 (CRTL1) *was also increased but to a lower extent (> 2-fold). The same expression pattern was detected for *collagen types IXα2 (COL9A2) *and *XIα1 (COL11A1)*, that expression was both significantly increased as the ND chondrocytes differentiated (> 4-fold). Also structural components of the cartilage ECM including *dermatopontin (DPT), asporin (ASPN), biglycan (BGN), cartilage intermediate protein 2 (CILP2), fibromodulin (FMOD), tenascin C (TNC) *and *fibronectin (FN1) *showed a significant increase in expression (3.3 to 67-fold) during 3D culture. The expression of different genes coding for ECM degrading enzymes, such as *a desintegrin and metalloproteinase with thrombospondin motifs (ADAMTS)-2 *(3.1-fold) and *matrix metalloproteinase (MMP)*-*2 *(1.9-fold), and *MMP7 *(109-fold), altogether involved in active matrix turnover of differentiating cells, was increased. On the contrary, the expression of *ADAMTS12 *(13-fold), *ADAMTS5 *(8-fold), and *MMP1 *(10-fold) was repressed while *tissue inhibitor of metalloproteinase (TIMP)-4 *(14-fold) was induced. Expression of growth factors including *insulin-like growth facto*r *(IGF)-1 *(8-fold) and *IGF2 *(40-fold) was highly increased. *TGF*-β1 (4-fold) and *bone morphogenetic protein (BMP)-1 *(2.1-fold) expression was increased to a lower extent and the same expression pattern could be detected for growth factor receptors including *TGFβ receptor 1 (TGFBR1) *and *fibroblast growth factor receptor 2 (FGFR2)*. Expression of a large number of transcription factors such as members of the *homeobox (HOX), SRY (sex determing region)-box (SOX), distal-less homeobox*, and *wingless-type MMTV integration site *gene families was induced during differentiation. Of special interest is the increased expression of *SOX9 *(4.4-fold), which acts as a direct regulator of *COL2A1 *expression. Another transcription factor that was found to be increased (> 4-fold) was *runt-related transcription factor 2 (RUNX2)*, known to be involved in several differentiation processes. Taken together, scaffold culture facilitated the induction of relevant marker genes for chondrogenic differentiation in ND chondrocytes.

**Table 3:** Classification of genes that are differentially expressed in chondrocyte monolayer (baseline) and scaffold culture

Functional annotation Gene title (Gene symbol)	Accession number	Fold change Scaffold vs Monolayer	
		
		Normal donors	OA donors
**Extracellular matrix**			
Aggrecan (ACAN)	[GenBank:X17406]	2.0	3.4 **
Asporin (ASPN)	[GenBank:NM_017680]	18.8 *	6.0 *
Biglycan (BGN)	[GenBank:NM_001711]	12.5	12.7 *
Cartilage intermediate layer protein 2 (CILP2)	[GenBank:BC034926]	78.2 *	71.3 *
Collagen, type II, alpha 1 (COL2A1)	[GenBank:X06268]	87.1	519.9 **
Collagen, type XI, alpha 1 (COL11A1)	[GenBank:BG028597]	10.4 **	5.9 *
Collagen, type IX, alpha 2 (COL9A2)	[GenBank:AI733465]	4.3	8.1
Cartilage link protein 1 (CRTL1)	[GenBank:NM_001884]	2.5	1.8
Cartilage oligomeric matrix protein (COMP)	[GenBank:NM_000095]	128.0 **	794.2 ***
Dermatopontin (DPT)	[GenBank:AL049798]	69.7 **	44.2 ***
Fibromodulin (FMOD)	[GenBank:NM_002023]	5.1	8.4 *
Fibronectin 1 (FN1)	[GenBank:AJ276395]	5.7 *	34.6 ***
TIMP metalloproteinase inhibitor 4 (TIMP4)	[GenBank:NM_003256]	14.1 *	25.4 *
Tenascin C (TNC)	[GenBank:BF434846]	3.3	3.5 *
**Cell adhesion and receptors**			
Epidermal growth factor receptor (EGFR)	[GenBank:AW157070]	-3.0 **	-1.9
Fibroblast growth factor receptor 2 (FGFR2)	[GenBank:NM_022969]	-7.4 *	-4.3
Laminin, alpha 2 (LAMA2)	[GenBank:AK026829]	5.7 *	4.8
Laminin, alpha 4 (LAMA4)	[GenBank:NM_002290]	-6.6 *	-8.6 **
Transforming growth factor, beta receptor I (TGFBR1)	[GenBank:AV700621]	4.2	20.0 ***
Thrombospondin 3 (THBS3)	[GenBank:L38969]	8.2 **	8.5 *
**Growth factors**			
Bone morphogenetic protein 1 (BMP1)	[GenBank:NM_001199]	2.1 *	1.8
Fibroblast growth factor 9 (FGF9)	[GenBank:NM_002010]	-9.0	-3.0
Insulin-like growth factor 1 (IGF1)	[GenBank:AI972496]	8.3	6.0
Insulin-like growth factor 2 (IGF2)	[GenBank:X07868]	114.9 **	43.5 **
Transforming growth factor, beta 1 (TGFB1)	[GenBank:BC000125]	3.0 *	2.4 *
**Transcription factors**			
Distal-less homeobox 5 (DLX5)	[GenBank:NM_005221]	5.1 *	25.6 *
Homeobox A11 (HOXA11)	[GenBank:H94842]	6.5	2.3 *
Homeobox A13 (HOXA13)	[GenBank:BG289306]	5.2	2.0
Runt-related transcription factor 2 (RUNX2)	[GenBank:AL353944]	4.0	4.2 *
SIX homeobox 1 (SIX1)	[GenBank:N79004]	1.6 *	1.3
SIX homeobox 4 (SIX4)	[GenBank:AI554514]	1.8	2.3
SRY (sex determining region Y)-box 9 (SOX9)	[GenBank:NM_000346]	4.4 **	11.8 **
Wingless-type MMTV integration site family, member 5B (WNT5B)	[GenBank:AW007350]	3.0	7.0
**Enzymes**			
ADAM metalloproteinase with thrombospondin type 1 motif, 12 (ADAMTS12)	[GenBank:W74476]	-13.7 **	-2.4
ADAM metalloproteinase with thrombospondin type 1 motif, 2 (ADAMTS2)	[GenBank:NM_021599]	3.1	4.7 **
ADAM metalloproteinase with thrombospondin type 1 motif, 5 (ADAMTS5)	[GenBank:BF060767]	-8.8 *	-7.6 **
Matrix metalloproteinase 1 (MMP1)	[GenBank:NM_002421]	-10.6 **	-59.7
Matrix metalloproteinase 2 (MMP2)	[GenBank:NM_004530]	1.9 *	3.4 *
Matrix metalloproteinase 7 (MMP7)	[GenBank:NM_002423]	109.7 ***	107.2 **

One hundred and seven genes associated with skeletal development and extracellular matrix formation were found differentially expressed in chondrocytes obtained from normal donors cultured in monolayer (baseline) and scaffolds. The expression patterns of these genes were compared with those of differentiating osteoarthritic (OA) chondrocytes to assess the chondrogenic capacity of these cells. Only genes are presented that belong to the shown functional categories. For the complete list see Additional data file 2. * *P *< 0.05; ** *P *< 0.01; *** *P *< 0.001.

### Gene expression analysis of chondrogenic potential of OA chondrocytes

The expression pattern of genes identified during ND chondrocyte differentiation was analyzed in cells obtained from patients with OA. Eighty five of the 107 genes significantly regulated during ND chondrocyte differentiation qualitatively displayed the same expression pattern during OA chondrocyte differentiation. *COL2A1 *was increased about 500-fold and *COMP *nearly 800-fold (Table 3) demonstrating a significantly higher increase in expression during differentiation compared with ND chondrocytes. Expression of other ECM components such as *COL9A2 *(8-fold) and *COL11A1 *(6-fold) as well as proteoglycans such as *biglycan *(12-fold), *dermatopontin *(44-fold), and *aggrecan *(3.4-fold) was also significantly upregulated as the OA cells differentiated (Table 3). As the expression profiles of OA and ND chondrocytes during differentiation do not completely overlap, OA-related differences were analyzed in more detail as described below.

### Gene expression analysis of OA and ND chondrocytes cultured in monolayer

Comparing monolayer cultures of OA and ND chondrocytes, expression of 32 genes related to skeletal development was detected as changed [see Additional data file 2]. Among them, *COMP *(6-fold), *FN1 *(3.1-fold), *TIMP3 *(2.1-fold), *TGFBR2 *(1.8-fold) and *SOX9 *(2.6-fold) were expressed at lower levels in OA chondrocytes, whereas *MMP1 *(5-fold) and *MMP3 *(2.6-fold), as well as the matrix components *COL5A3 *(2.9-fold), *COL3A1 *(2.2-fold) and *periostin *(1.9-fold) displayed an increased expression in OA chondrocytes (Table 4).

**Table 4:** Genes differentially expressed comparing chondrocytes in culture obtained from osteoarthritic (OA) and normal donors (ND)

Functional annotation Gene title (Gene symbol)	Accession number	Fold change	Signal	
		**OA vs ND**	**OA**	**ND**

**Monolayer**				
ADAM metalloproteinase with thrombospondin type 1 motif, 1 (ADAMTS1)	[GenBank:AF060152]	-1.7	968.0	1925.2
Collagen, type III, alpha 1 (COL3A1)	[GenBank:AU146808]	2.2	564.3	258.7
Collagen, type V, alpha 3 (COL5A3)	[GenBank:AI984221]	2.9	977.6	392.1
Collagen, type XI, alpha 1 (COL11A1)	[GenBank:BG028597]	1.9	327.8	161.1
Cartilage link protein 1 (HAPLN1)	[GenBank:NM_001884]	1.8 *	2640.7	1467.5
Cartilage oligomeric matrix protein (COMP)	[GenBank:NM_000095]	-6.1 *	24.7	187.4
Dystonin (DST)	[GenBank:BC004912]	-2.0	729.4	1486.6
Fibronectin 1 (FN1)	[GenBank:AJ276395]	-3.1	441.9	1642.6
Matrix metalloproteinase 1 (MMP1)	[GenBank:NM_002421]	5.0 *	857.0	200.5
Matrix metalloproteinase 2 (MMP2)	[GenBank:NM_004530]	-1.9	2300.1	3706.1
Matrix metalloproteinase 3 (MMP3)	[GenBank:NM_002422]	2.6	2496.8	1052.8
Periostin, osteoblast specific factor (POSTN)	[GenBank:AY140646]	1.9	6610.8	3544.5
SRY (sex determining region Y)-box 9 (SOX9)	[GenBank:AI382146]	-2.6 *	123.0	281.2
Transforming growth factor, beta receptor II (TGFBR2)	[GenBank:D50683]	-1.8	970.0	1760.5
TIMP metalloproteinase inhibitor 3 (TIMP3)	[GenBank:NM_000362]	-2.1	524.5	1067.1
**Scaffold**				
Collagen, type VI, alpha 1 (COL6A1)	[GenBank:BE350145]	1.6	1125.6	767.4
Collagen, type VIII, alpha 2 (COL8A2)	[GenBank:AI806793]	-1.5	749.8	1067.1
Catenin, beta 1 (CTNNB1)	[GenBank:AF130085]	1.8	1254.4	785.5
Dystonin (DST)	[GenBank:BC004912]	1.8	1969.3	1178.2
Fibulin 1 (FBLN1)	[GenBank:Z95331]	-1.3	557.5	728.4
Fibronectin 1 (FN1)	[GenBank:W73431]	3.5	1160.8	410.2
Homeobox A13 (HOXA13)	[GenBank:BG289306	-2.1	30.5	62.8
Homeobox C6 (HOXC6)	[GenBank:NM_004503]	1.8	857.2	525.7
Latent transforming growth factor beta binding protein 1 (LTBP1)	[GenBank:AI986120]	1.6	997.6	646.6
Myocyte enhancer factor 2C (MEF2C)	[GenBank:N22468]	1.7 *	498.1	296.5
Microfibrillar-associated protein 2 (MFAP2)	[GenBank:NM_017459]	-1.4	2875.4	4083.6
Tissue factor pathway inhibitor 2 TFPI2)	[GenBank:AL574096]	2.8	76.2	38.3
Transforming growth factor, beta receptor I (TGFBR1)	[GenBank:AV700621]	2.6	682.7	303.9
TIMP metalloproteinase inhibitor 3 (TIMP3)	[GenBank:BF347089]	-1.5	216.2	308.3
WNT1 inducible signaling pathway protein 3 (WISP3)	[GenBank:AF143679]	-2.0	318.5	549.8

Genes were functionally filtered with regard to their association with skeletal development and extracellular matrix formation. For the complete list see Additional data file 1. * *P *< 0.05; ** *P *< 0.01; *** *P *< 0.001.

### Gene expression analysis of OA and ND chondrocytes cultured in Hyaff-11 scaffolds

In scaffold cultures, only 17 genes related to differentiation and ECM were differentially expressed. Among those genes, which were already discussed, only *FN1 *(1.8-fold), *dystonin (DST) *(3.5-fold), and *TIMP3 *were still differentially expressed; however, expression of *FN1 *and *DST *was reversed compared with ML (Table 4). Altogether, the differences detected between OA and ND chondrocytes cultured in ML were further diminished as the cells differentiated in Hyaff-11 scaffolds.

**Figure 4:**
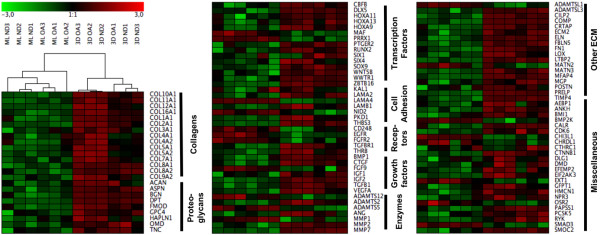
Hierarchical cluster analysis of chondrocytes from osteoarthritic and normal donors cultured in monolayer and Hyaff-11 scaffolds.  Genes that were differentially expressed between normal donors (ND) chondrocytes cultured in monolayer (ML) and scaffold (3D) cultures, functionally filtered by their association with skeletal development and extracellular matrix (ECM) formation, were used to assess chondrogenic capacity of chondrocytes from osteoarthritic (OA) patients. Green bars depict a repressed and red bars an induced expression of genes normalized to the mean. The clustering gave two main groups classified as monolayer chondrocytes and scaffold-cultured chondrocytes. The separate OA monolayer cluster clearly indicated a differential expression pattern between OA and ND chondrocytes. In scaffold cultures on the other hand, no OA-related cluster separation was observed demonstrating a loss of differences between OA and ND chondrocytes during scaffold culture.

Considering the expression pattern of ND chondrocytes, hierarchical clustering resulted in two main groups, classified as ML and scaffold (Figure 4). The clustering also showed that the ML-cultured OA and ND chondrocytes clustered, while no such clustering was detected in cells cultured in Hyaff-11 culture. Additionally, the total number of genes (without functional filtering) differentially expressed between OA and ND chondrocytes was remarkable reduced in scaffold culture (184) in comparison with ML (661 genes; Table 2) [see Additional data file 1].

### PCR validation of microarray results

**Figure 5:**
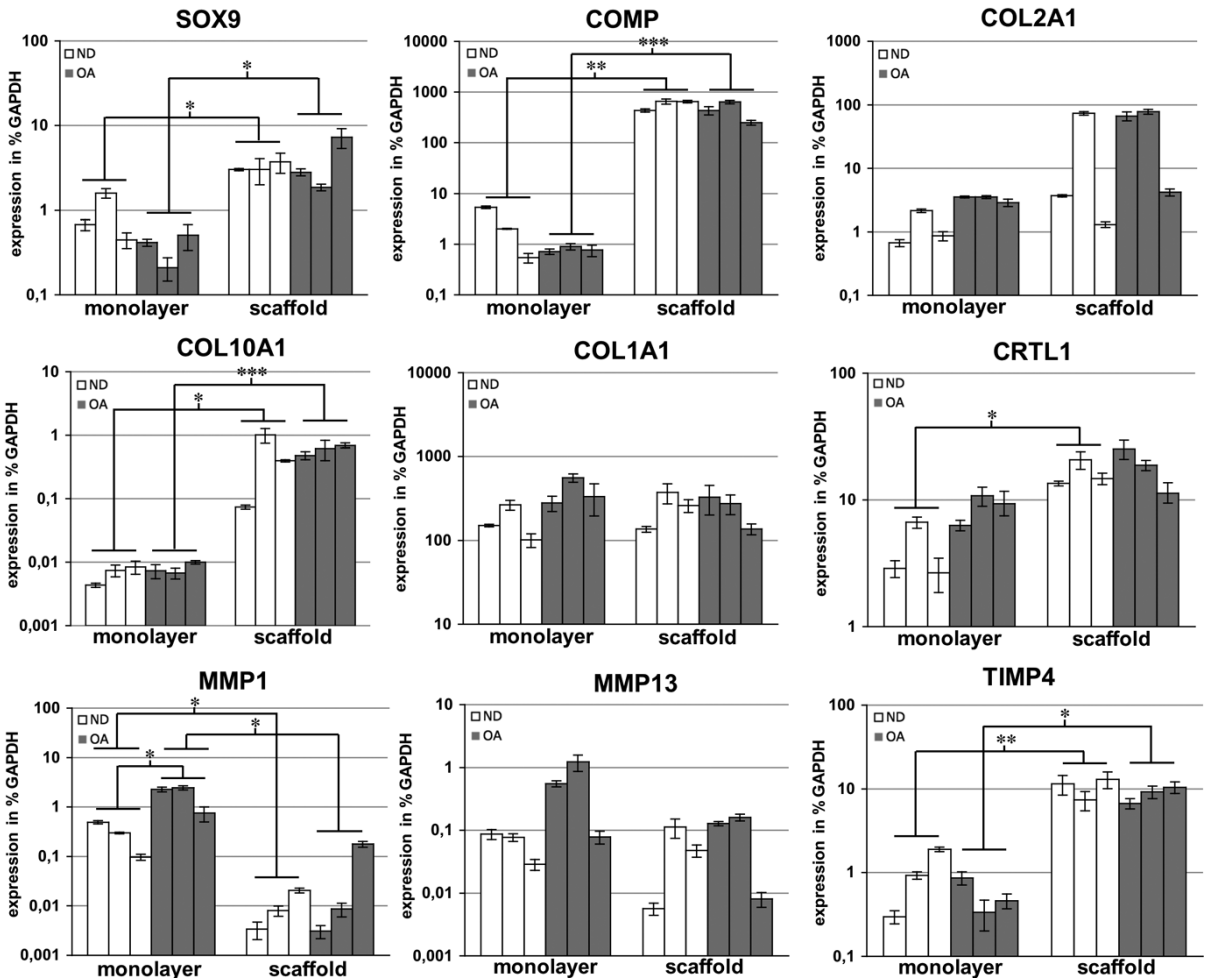
Real-time PCR verification of results from the microarray analysis.  The expression was calculated as percentage of the expression of the housekeeping gene glyceraldehyde-3-phosphate dehydrogenase (GAPDH). The mean of each technical triplicate is plotted and the error bars represent standard deviation. * P < 0.05; ** P < 0.01; *** P < 0.001.

In order to confirm expression profiles as assessed by microarray analysis, the expression of selected genes was analyzed by real-time PCR (Figure 5). Expression of the cartilage markers *COMP *and *SOX9 *was found to be highly induced during scaffold culture, as also seen in the microarray analysis. *COL2A1 *and *CRTL1 *were also highly expressed in scaffold culture but with more donor-dependent variations. *COL10A1 *expression, associated with cartilage hypertrophy, was also increased during scaffold culture, but no difference between OA and ND chondrocytes was detected. In contrast, the expression of *MMP1 *was higher in OA chondrocytes cultured in ML compared with ND chondrocytes. The expression of this gene was then significantly reduced in scaffold culture in both groups of donors to a comparable level. No significant differences in expression of *MMP13 *and *COL1A1 *were detected comparing cells cultured in ML or scaffolds as well as comparing OA and ND chondrocytes. Taken together, PCR analysis demonstrated the same gene expression pattern as the microarray analysis in all nine genes analyzed by real-time PCR.

## Discussion

In order to be able to use second-generation ACT techniques for the repair of cartilage defects in patients with OA, it is highly important to investigate whether OA chondrocytes have an irreversibly altered phenotype or if these cells can differentiate towards a hyaline cartilage phenotype after *in vitro *expansion. Today, there are conflicting data whether OA chondrocytes fulfill the prerequisites for ACT treatment or not [12,13,15,21]. This encouraged us to investigate more thoroughly the chondrogenic differentiation potential of human OA chondrocytes using microarray technology in order to determine whether OA chondrocytes might possibly be used in second-generation ACT.

Microarray analysis of human OA and ND chondrocytes cultured in ML indicated that the OA chondrocytes were in a less differentiated state compared with the ND chondrocytes. This is thus in accordance with the differences detected *in vivo *between OA and ND cartilage [10,22]. Re-differentiation in scaffold cultures diminished these differences, demonstrating that only 17 genes related to skeletal development were significantly differentially expressed between both groups. This high similarity was not only detected on gene expression level but also in their ability to accumulate sulphated proteoglycans and collagen type II, matrix components characteristic for a hyaline cartilage phenotype. High-density pellet cultures confirmed these results, demonstrating differentiation towards the hyaline cartilage lineage for both ND and OA chondrocytes. Differentiation in the scaffolds was for both ND and OA chondrocytes associated with significantly increased expression of matrix constituents characteristic for mature articular cartilage, including *aggrecan, biglycan, CILP2, COL2A1*, *COL9A2*, *COL11A1*, *COMP*, and *FN1 *[23-27]. Another sign of chondrogenic differentiation was the increased expression of *TGFB1 *as well as *DPT*, which have been demonstrated to increase the cellular response to TGFβ [28,29]. In contrast, *COMP*, *FN1*, and *SOX9 *displayed a reduced expression while *COL3A1*, *MMP1 *and *MMP3 *showed increased expression in OA chondrocytes compared with ND chondrocytes cultured in ML. Except for *TIMP3*, no significant differences were consistently detected between OA and ND chondrocytes after 14 days of re-differentiation in scaffolds considering a gene set relevant for differentiation.

An increased expression of the hypertrophic cartilage marker *COL10A1 *gene has been reported in OA cells in comparison to normal chondrocytes, which might limit their use in tissue engineering [11]. However, our results did not demonstrate a significant difference in the expression of *COL10A1 *between normal and OA chondrocytes in scaffold culture, neither did we detect any differences in the expression of markers for endochondral bone formation including *alkaline phosphatase, parathyroid hormone receptors 1 *and *2, periostin and RUNX2 *[30-33]. The induction of genes such as *COL10A1 *and *RUNX2 *in our scaffold cultures is primarily caused by the use of the chondrogenic factor TGF-β1, which was also observed in chondrogenically induced micromasses of chondrocytes or mesenchymal stem cells [34-36]. This model-inherent *COL10A1 *induction does not inhibit the detection of different *COL10A1 *expression levels as shown by Tallheden and colleagues [15], and maybe can be inhibited by the addition of factors such as parathyroid hormone-related protein [37]. Accordingly, the risk of differentiation into the hypertrophic cartilage lineage thus does not seem to be increased for the OA chondrocytes. In accordance with our results, Stoop and colleagues recently demonstrated that ML expanded normal and OA chondrocytes transplanted subcutaneously into immunodeficient mice for eight weeks displayed no significant differences in their expression of *aggrecan, COL1A1*, *COL2A1*, or *COL10A1 *[14]. Our results further demonstrate that the expression of matrix proteins characterizing the phenotypical alteration of OA chondrocytes, that is, increased expression of *COL1A1*, *COL3A1*, *TNC *[38-40], did not display a significantly higher expression in OA chondrocytes compared with normal chondrocytes, either after ML culture or in scaffolds. This suggests that the cells have already acquires a normal phenotype after the second passage. These results are in accordance with Yang and colleagues, who demonstrated diminishing differences on mRNA level from passage 1 to 2 between normal and OA chondrocytes [41]. The same results were obtained for several *MMPs*, *TIMPs*, and *ADAMs *that are differentially regulated between OA and normal cartilage [42,43]. Interestingly, we detected that *MMP13*, which is the principal degradative enzyme for collagen types I, II and III in OA [44], has a somewhat altered expression in OA chondrocytes than in the ND chondrocytes after expansion, but this difference was diminished after re-differentiation. Likewise, hierarchical clustering analysis of genes relevant for differentiation demonstrated diminishing differences in gene expression from ML to differentiation in scaffolds, further suggesting that differences between OA and ND chondrocytes are decreased during re-differentiation in scaffolds. We thus conclude that after expansion and re-differentiation the chondrocytes from OA patients are not significantly different from those from normal donors used in ACT. At this point, we want to point out that mRNA expression do not always reflect protein secretion. Nevertheless, 3D culture in high-density micromasses or scaffolds seem to be appropriate to further stabilize the chondrocyte phenotype without additional manipulation of the cells.

Some of the differentially expressed genes in ML seem to be OA-related, and thus may serve as OA indicators *in vitro*. Especially genes coding for secreted proteins such as ECM components, growth factors and degradative enzymes could be of interest for the establishment of a non-destructive detection assay to ensure cell and culture quality, identity and purity. Increased expression of *MMP1 *and *MMP3 *[45] as well as *type III and type V collagen *expression [46] has been shown to be associated with OA-related cartilage destruction. In contrast, an induction of *MMP13 *and *COL2A1 *during OA progression in native cartilage was described [47,48], but both genes were not found to be differentially expressed in ML in our study, and thus seem not to be appropriate to distinguish between normal and OA chondrocyte subcultures.

In this study, an estrified hyaluronic acid-based scaffold was used as a vehicle for re-differentiation of the chondrocytes because it has been used successfully in matrix-assisted cartilage repair in combination with culture-expanded chondrocytes [7,49]. Compared with pellet cultures, scaffold cultures demonstrated a less differentiated phenotype on protein level after 14 days of *in vitro *culture, but on gene expression level the typical pattern of chondrogenic differentiation was detected indicating a delayed differentiation in scaffold culture. This delay is not necessarily disadvantageous in clinical practice. One important factor in ACT is the integration of the scaffold to the surrounding cartilage and subchondral bone. It was recently demonstrated by Obradovic and colleagues that integration of the scaffold is dependent on the differentiation grade of the cells [50]. Less differentiated tissue engineered cartilage implants (re-differentiated for five days) demonstrated better integration properties compared with mature implants (re-differentiated for five weeks). The fact that the cells were less differentiated in the scaffolds might thus be an advantage for its clinical use. In clinical practice of Hyaff-11 scaffolds, prior ACT treatment the chondrocytes are differentiated for 14 days and attain a differentiation grade similar to the one obtained in our cultures. Other clinically matrix-associated ACT techniques also made use of seeding less differentiated chondrocytes with comparable outcome further confirming that transplantation of less differentiated chondrocytes is not a disadvantage [3,5,6].

One important issue, that in the context of a clinical application needs to be further investigated, is the impact of the inflammatory environment in OA cartilage on the transplanted chondrocytes. Cytokines, including IL-1 and TNF-α, are secreted by OA cartilage. Such cytokines are known to induce cartilage degradation and to reduce collagen type II expression [51]. These factors might not only degrade articular cartilage, but may also affect the transplant. To ensure good clinical results, it seems to be highly important to control the inflammatory environment, for example by treating the patient with cytokine inhibitors as well as removing any degenerated cartilage surrounding the defect.

The emerging interest of cell-based regeneration of OA has lead to the first systematic clinical considerations. Hollander and colleagues reported tissue regeneration when tissue engineered cartilage was implanted in injured and OA human knees [21]. Ossendorf and colleagues performed a study on short-term and mid-term efficacy of second-generation ACT for treatment of degenerative cartilage defects [5]. Both suggested that this technique is an effective treatment option for the regeneration of OA defects of the knee, and OA does not inhibit the regeneration process.

Our findings were made on a rather small individual basis comparing only a few individuals per group. We encountered this lack of statistical power by having well-defined groups of cartilage donors (Mankin and Ahlbäck Scores, ND age matching), and by applying detection techniques sensitive to identify subtle distinctions in chondrogenic capacity (genome-wide microarray analysis, PCR, Bern Score). NDs meet the demands of patients undergoing ACT treatment for isolated articular lesions without further indications such as OA or inflammatory diseases, in accordance with widely accepted treatment guidelines [52]. ACT treatment is a method to repair focal cartilage lesions, which can occur as a result of traumatic mechanical destruction. Chondrocytes from these patients might thus slightly differ from chondrocytes obtained from healthy joints. [53]. However, ACT patient-derived chondrocytes represent an ideal baseline when thinking about a further development of ACT for treatment of OA patients. The OA donors underwent hip replacement surgery and were accordingly older (12 years in average age) than the ND donors. Barbero and colleagues identified age-dependent differences in the chondrogenic ability of expanded chondrocytes applying the pellet assay. The chondrogenic capacity was decreased in donors over 40 years of age compared with younger ones, but no further significant decline of chondrogenic ability with increasing age was observed [54], so that the difference in age can be neglected. To ensure that even subtle distinctions between ND and OA chondrocytes were detected, relaxed selection criteria in comparative gene expression analysis were applied. Thorough statistical analysis was performed to reveal the significance level. Importantly, large-scale gene expression analysis was performed applying a clinical relevant model and appropriate settings. Therefore, our study provides useful information, which is important for chondrocyte-based cartilage repair procedures, especially in the discussion whether chondrocyte differentiation potential is independent of OA etiology or not.

## Conclusions

Gene expression profiling indicated that chondrocytes from OA donors showed a less differentiated state in ML compared with ND chondrocytes. During 3D culture in scaffolds, the differences in gene expression between OA and ND chondrocytes were diminished. Differences in expression of markers for hypertrophic cartilage were not observed. Thus, OA chondrocytes show a chondrogenic differentiation potential comparable with ND chondrocytes and the risk of differentiation into the hypertrophic cartilage lineage thus does not seem to be increased. Our findings suggest that chondrocytes from human OA cartilage fulfill the prerequisite for use in matrix-assisted ACT.

## Abbreviations

3D: three-dimensional; ACAN: aggrecan; ACT: autologous chondrocyte transplantation; ADAMTS: a disintegrin and metalloproteinase with thrombospondin motifs; ASPN: asporin; BGN: biglycan; BMP: bone morphogenetic protein; BSA: bovine serum albumin; CILP2: cartilage intermediate layer protein 2; COL1A1: collagen type Iα1; COL2A1: collagen type IIα1; COL3A1: collagen type IIIα1; COL9A2: collagen type IXα3; COL10A1: collagen type Xα1; COL11A1: collagen type XIα2; COMP: cartilage oligomeric matrix protein; CRTL1: cartilage link protein 1; DMEM: Dulbecco's Modified Eagle Medium; DPT: dermatopontin; DST: dystonin; ECM: extracellular matrix; FC: fold change; FGFR: fibroblast growth factor receptor; FMOD: fibromodulin; FN1: fibronectin 1; GAPDH: glyceraldehyde-3-phosphate dehydrogenase; HOX: homeobox; IGF: insulin-like growth factor; IL: interleukin; ML: monolayer; MMP: matrix metalloproteinase; ND: normal/healthy donor; OA: osteoarthritis; PBS: phosphate-buffered saline; PCR: polymerase chain reaction; RUNX2: runt-related transcription factor; SOX: SRY (sex determining region Y)-box; TGF: transforming growth factor; TIMP: tissue inhibitor of metalloproteinase; TNC: tenascin C; TNF: tumor necrosis factor.

## Competing interests

MS works as a consultant for BioTissue Technologies GmbH (Freiburg, Germany). This company develops autologous tissue transplants for the regeneration of bone and cartilage. He is also shareholder of CellServe GmbH (Berlin, Germany) and BioRetis GmbH (Berlin, Germany). The product activities of both companies have no connection with the topics discussed here. AL is a shareholder in Cell Matrix. This company develops and produces transplantation products and laboratory services for cartilage cell therapy. JR, TD, and CK declare they have no competing interests.

## Authors' contributions

CK and TD carried out the gene expression data processing, participated in the design and coordination of the study and drafted the manuscript. JR participated in gene expression data processing, study design and coordination. AL and MS conceived the study and participated in its design and coordination. All authors read and approved the final manuscript.

## Supplementary Material

Additional file 1 Excel file containing a table of microarray expression data of comparative gene expression analysis. A total list of genes differentially expressed between cultures of chondrocytes obtained from osteoarthritic (OA) and normal donors (ND) is given. Group comparisons were performed between: scaffold and monolayer cultures from ND chondrocytes; scaffold and monolayer cultures from OA chondrocytes; chondrocytes from OA and ND cultured in monolayer; and scaffolds cultures (see to Figure 1 for experimental setup). Expression differences are given as fold change (FC) with monolayer or normal donor as baseline. Click here for file

Additional file 2 Excel containing a table of microarray expression data of genes relevant for differentiation. Listed are genes that were differentially expressed in cultured chondrocytes obtained from osteoarthritic (OA) and normal donors (ND) resulting from following comparisons: scaffold and monolayer cultures from ND chondrocytes; scaffold and monolayer cultures from OA chondrocytes; chondrocytes from OA and ND cultured in monolayer; and scaffolds cultures. Genes were functionally filtered by annotations of the Gene Ontology Database (search terms 'skeletal development' and 'extracellular matrix formation'). Expression differences are given as fold change (FC). Either monolayer or ND was set as baseline. Click here for file
